# Retraction: A versatile strategy for alternately arranging the foam ratio layers of multilayer graphene/thermoplastic polyurethane composite foams towards lightweight and broadband electromagnetic wave absorption

**DOI:** 10.1039/d1ra90101j

**Published:** 2021-04-15

**Authors:** Chaozhi Wang, Jiang Li, Shaoyun Guo

**Affiliations:** The State Key Laboratory of Polymer Materials Engineering, Polymer Research Institute of Sichuan University Chengdu 610065 China li_jiang@scu.edu.cn +86-28-85466077 +86-28-85466077

## Abstract

Retraction of ‘A versatile strategy for alternately arranging the foam ratio layers of multilayer graphene/thermoplastic polyurethane composite foams towards lightweight and broadband electromagnetic wave absorption’ by Chaozhi Wang *et al.*, *RSC Adv.*, 2019, **9**, 23843–23855, DOI: 10.1039/C9RA04405A.

We, the named authors, hereby wholly retract this *RSC Advances* article due to concerns affecting the reliability of the data.

In Fig. 8(b) and (c), the layer interface is not the real layer interface, because the layer interfaces of S-3 and S-5 were combined using Photoshop technology. Fig. 8(b) was created from 2 different photos, and Fig. 8(c) was created from 3 different photos. So, the thickness data of each layer is not the real thickness data.

In Fig. 6, characteristic impedance (a) and attenuation constant (b) were calculated by eqn (13) and (14). However, *μ*′ and *μ*′′ should be taken as 1 and 0, respectively, because of the weak magnetic properties of TPU/graphene composites. Therefore, eqn (13) and (14) in the article are incorrect and should have been replaced by the following equations.
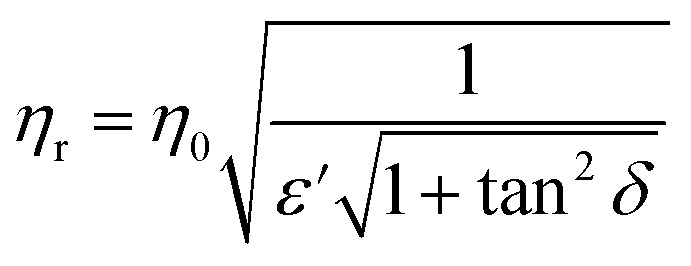

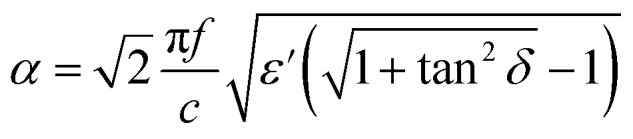


On page 23848 of the article, it states “The high conductivity was produced by the three-dimensional conductive network structure, which was induced by graphene’s selective dispersion and alignment in the pore structures, as can be observed in Fig. 5b”. However, in Fig. 5b, the three-dimensional conductive network structure and graphene’s selective dispersion and alignment in the pore structures cannot be seen very clearly, because it is only a cell structure diagram, and therefore does not prove the existence of a three-dimensional conductive path.

As the above data errors and misuse of pictures have greatly influenced the reliability of the conclusions, we are retracting this article to protect the integrity and accuracy of the scientific record.

 

Signed: Chaozhi Wang, Jiang Li and Shaoyun Guo

Date: 5^th^ April 2021

 

Retraction endorsed by Laura Fisher, Executive Editor, *RSC Advances*

## Supplementary Material

